# Sca1 marks a reserve endothelial progenitor population that preferentially expand after injury

**DOI:** 10.1038/s41421-021-00303-z

**Published:** 2021-09-28

**Authors:** Juan Tang, Huan Zhu, Shaoyan Liu, Haixiao Wang, Xiuzhen Huang, Yan Yan, Lixin Wang, Bin Zhou

**Affiliations:** 1 The State Key Laboratory of Cell Biology, CAS Center for Excellence on Molecular Cell Science, Shanghai Institute of Biochemistry and Cell Biology, Chinese Academy of Sciences, University of Chinese Academy of Sciences, Shanghai, China; 2 Zhongshan Hospital, Fudan University, Shanghai, China; 3 School of Life Science and Technology, ShanghaiTech University, Shanghai, China; 4 School of Life Science, Hangzhou Institute for Advanced Study, University of Chinese Academy of Sciences, Hangzhou, China; 5 Institute for Stem Cell and Regeneration, Chinese Academy of Sciences, Beijing, China

**Keywords:** Adult stem cells, Regeneration

Dear Editor,

Vascular endothelial cell renewal, repair and regeneration are critical for tissue homeostasis and response to injuries^1^. Unraveling the heterogeneity and hierarchy of endothelial cells in homeostasis and after injuries provides valuable information of potential targets for therapeutic neovascularization. Stem cell antigen-1 (Sca1) is a member of the ly-6 family, which was reported as cell surface markers of hematopoietic stem cells^2^. Sca1^+^ progenitor cells residing in the heart do not contribute to cardiomyocytes, but instead adopt vascular endothelial cell fate^3–5^. Whether Sca1-expressing cells represent a unique population of endothelial cells (ECs) that differs from other Sca1^–^ ECs remains largely unknown. In addition, whether the Sca1 expression heterogeneity in ECs may indicate the existence of a functional hierarchy for endothelial cells in angiogenesis is unclear. We first isolated CD31^+^ endothelial cells by FACS and performed single-cell RNA sequencing (scRNA-seq). Uniform manifold approximation and projection (UMAP) analysis of this dataset revealed clusters of endothelial and adventitial cell populations based on marker gene expression (Supplementary Fig. S1a, b), with Ly6a (Sca1) marking different populations of ECs and makers for others subtype cells such as pericytes, endocardial cells and fibroblasts (Supplementary Fig. S1b, c). Further pathway enrichment analysis showed that cell proliferation and angiogenesis-related pathways were highly enriched in Sca1^high^ ECs compared with the Sca1^low^ ECs (Supplementary Fig. S1d), indicating these Sca1^high^ ECs may exhibit specific functions during cardiac homeostasis and after injuries. Next we generated *Sca1-2A-CreER;R26-GFP*^6^ to lineage trace Sca1^+^ cells in the adult tissues during homeostasis and after injury (Fig. 1a). We collected heart samples at 24–48 hours after tamoxifen induction (Fig. 1b). Immunostaining for GFP, VE-Cad or PECAM showed that 30.54 ± 1.01% of VE-Cad^+^ and 31.90 ± 0.67% of PECAM^+^ ECs express GFP (Fig. 1c, d). We confirmed that ~30% of ECs were GFP^+^ by FACS analysis of heart ventricles (Fig. 1e). We next examined the heart tissues at 12 to 16 weeks after tamoxifen treatment (Fig. 1f). By immunostaining and FACS analysis of heart ventricles, we did not find a significant increase of GFP percentage in ECs after 12–16 weeks’ tracing (Fig. 1g–i). EdU incorporation assays showed there was no significant difference of the percentage of EdU^+^ ECs between GFP^–^ and GFP^+^ EC populations (Fig. 1j). These data indicate that Sca1^+^ ECs proliferate at the similar rate as Sca1^–^ ECs at homeostasis.

**Fig. 1:**
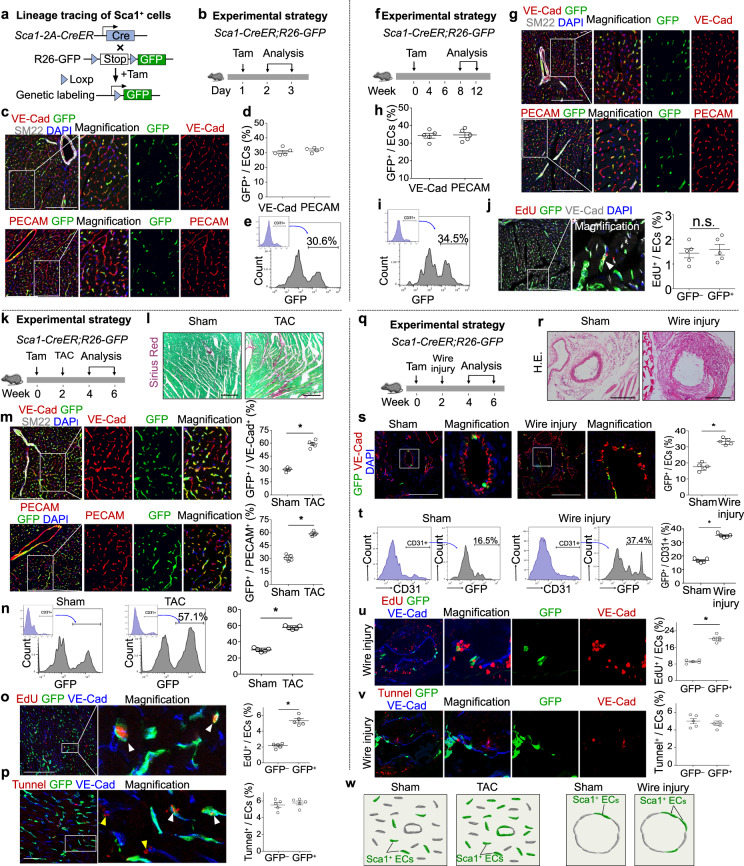
Sca1^+^ endothelial cells expand preferentially after injuries. **a** Schematic figure showing genetic lineage tracing of Sca1^**+**^ cells. **b** Schematic figure showing experimental design. **c** Immunostaining for GFP, VE-Cad, SM22, or PECAM on heart sections. **d** Quantification of the percentage of VE-Cad^**+**^ or PECAM^**+**^ endothelial cells (ECs) expressing GFP. **e** Flow cytometric analysis of CD31^**+**^ ECs expressing GFP. **f** Schematic diagram showing experimental design for homeostasis study. **g** Immunostaining for GFP, VE-Cad, SM22, or PECAM on heart sections. **h** Quantification of the percentage of VE-Cad^**+**^ or PECAM^**+**^ ECs expressing GFP. **i** Flow cytometric analysis and quantification of the percentage of CD31^**+**^ cells expressing GFP. **j** Immunostaining for EdU, GFP, and VE-Cad on heart sections. Quantification of the percentage of EdU^**+**^ cells in GFP^+^ or GFP^–^ populations. **k** Schematic diagram showing experimental design for TAC study. **l** Sirius red staining on sections from sham and TAC hearts. **m** Immunostaining for GFP, VE-Cad, SM22, or PECAM on heart sections, and the quantification of the percentage of VE-Cad^+^ or PECAM^+^ ECs expressing GFP. **n** Flow cytometric analysis and quantification of the percentage CD31^+^ ECs expressing GFP. **o** Immunostaining for EdU, GFP, and VE-Cad on TAC heart sections. Quantification of the percentage of GFP^–^ or GFP^+^ ECs incorporating EdU. **p** Immunostaining for TUNEL, GFP and VE-Cad on TAC heart sections. Quantification of the percentage of GFP^–^ or GFP^+^ ECs that are TUNEL^+^. **q** Schematic diagram showing experimental design for wire-induced injury. **r** HE staining on artery sections from sham or wire injury groups. **s** Immunostaining for GFP and VE-Cad on sections. Quantification of the percentage of ECs expressing GFP. **t** Flow cytometric analysis and quantification of the percentage of CD31^+^ ECs expressing GFP. **u** Immunostaining for EdU, GFP, and VE-Cad on injury arteries. Quantification of the percentage of GFP^–^ or GFP^+^ ECs incorporating EdU. **v** Immunostaining for TUNEL, GFP, and VE-Cad on injury arteries. Quantification of the percentage of GFP^–^ or GFP^+^ ECs that are TUNEL^+^. **w** Cartoon image showing preferential expansion of Sca1^+^ ECs after TAC or wire induced artery injuries. Scale bars, 100 µm. Data are means ± SEM; *n* = 5; *P < 0.05; ns, non-significant. Each figure is representative of 5 individual biological samples.

We next exposed *Sca1-2A-CreER;R26-GFP* mice to cardiac stress by transverse aortic constriction (TAC) model at two weeks after tamoxifen treatment (Fig. 1k). Sirius red staining on heart sections showed more fibrosis after TAC (Fig. 1l). Immunostaining for GFP, VE-Cad or PECAM on tissue sections showed a significant increase of the percentage of GFP^+^ ECs in TAC group compared with Sham (Sham 29.34 ± 0.98% vs TAC 59.12 ± 1.85% of VE-Cad^+^ ECs; Sham 30.82 ± 1.48% vs TAC 58.64 ± 1.04%, Fig. 1m). Flow cytometric analysis of left ventricle confirmed that the percentage of GFP^+^ ECs in TAC was significantly higher than sham group (Sham 30.05 ± 1.02% vs TAC 57.69 ± 0.96%, Fig. 1n). By detection of EdU incorporation, we found more GFP^+^ ECs incorporate EdU than GFP^–^ ECs after TAC (Fig. 1o). This result was confirmed by Ki67 and pHH3 staining (Supplementary Fig. S2a, b). In addition, there was no significant difference in cell death between Sca1^+^ and Sca1^–^ ECs in post-injury hearts (Fig. 1p). Taken together, these data demonstrate that Sca1^+^ ECs respond to cardiac stress and expand more preferentially after injury.

To examine if Sca1^+^ ECs in the large arteries are also unique in cell proliferation compared with Sca1^–^ ECs, we performed wire-induced femoral artery injury model on *Sca1-2A-CreER;R26-GFP* mice at two weeks after tamoxifen treatment (Fig. 1q). HE staining showed neointimal formation, indicating successful wire-induced vessel injury (Fig. 1r). Immunostaining for GFP and VE-Cad on tissue sections showed a significant increase of the percentage of GFP^+^ ECs after wire-induced injury compared with sham group (Sham 17.63 ± 1.07% vs Wire injury 33.37 ± 0.88%, Fig. 1s). Flow cytometric analysis showed that a significant increase of GFP^+^ ECs percentage after wire injury (Sham 16.66 ± 0.62% vs Wire injury 34.97 ± 0.55%, Fig. 1t). By EdU incorporation analysis, we found the percentage of GFP^+^ ECs incorporating EdU was significantly higher than that of GFP^–^ ECs after wire injury (Fig. 1u). In addition, the percentage of GFP^+^ ECs expressing Ki67 or pHH3 was significantly higher than that of GFP^–^ ECs in injured arteries (Supplementary Fig. S2c, d). We did not detect any significant difference in cell death between Sca1^+^ and Sca1^–^ ECs after wire injury (Fig. 1v). Taken together, Sca1^+^ ECs in the large arteries also respond and expand preferentially after injury.

This study reported that Sca1^+^ ECs represent a distinct EC sub-population that preferentially expand after TAC or wire injury (Fig. 1w). Recently, a number of reports have suggested that Sca1^+^ multipotent stem cells can give rise to endothelial cells. Qu-Petersen et al. showed that Sca1^+^ skeletal muscle-derived stem cells are able to differentiate into endothelial cells, and contribute to the regeneration of the skeletal muscle in a murine model of Duchenne’s muscle dystrophy^7^. Myocardium-derived adult Sca1^+^ cells isolated by cell sorting techniques were also capable of differentiating into endothelial cells^8–10^. However, there is no direct in vivo evidence supporting the existence of Sca1^+^ side population cells for endothelial cell contribution during tissue homeostasis and after injury. In our study, we used *Sca1-CreER* to fate-map Sca1^+^ cells in tissue homeostasis and after injuries. By scRNA-seq, we found cell proliferation or cell cycle regulation and angiogenesis-related pathways were highly enriched in Sca1-high ECs compared with that of Sca1-low ECs, indicating the potential higher cell proliferation and angiogenesis-related function of these cells (Supplementary Fig. 1d). While we found that there is no significant difference of EC renewal between Sca1^+^ and Sca1^−^ EC populations under homeostasis by EDU/Ki67 and pHH3 immunostaining. Consistent with results in heart after TAC injury, we found the percentage of Sca1^+^ ECs that have incorporated EdU was significantly higher than that from Sca1^–^ ECs after wire injury. These data suggested that Sca1^+^ ECs have stronger proliferation ability, which is activated after injury. The detection of cell proliferation difference in scRNA-seq but not by immunostaining during homeostasis could be explained by the sensitivity of the two different techniques in measuring gene expression. Our fate mapping results suggest that Sca1^+^ cells represent a reserve endothelial cell population that preferentially expands after injuries. Our study also supported the existence of an endothelial cell hierarchy within tissue, providing new avenues for future therapeutic interventions for vascular diseases. While Sca1^+^ endothelial progenitors give rise to more endothelial cells after injuries, whether other endothelial cell populations or progenitors contribute to new endothelial cells need further studies. Future studies would be required to unravel the underlying mechanism for contribution of Sca1^+^cells to more endothelial cells in vascular repair and regeneration.

## Supplementary information

Suppl. Info.
