# Supplementary material for: Sca1 marks a reserve endothelial progenitor population that preferentially expand after injury
Source: Cell Discov. 2021 Sep 28;7:88. doi: 10.1038/s41421-021-00303-z (PMC8476504; doi:10.1038/s41421-021-00303-z)
Supplement: Supplementary file 1 — Suppl. Info. [file 41421_2021_303_MOESM1_ESM.pdf]

Supplementary Information

Sca1 marks a reserve endothelial progenitor population that preferentially expand after injury

Juan Tang, Huan Zhu, Shaoyan Liu, Haixiao Wang, Xiuzhen Huang, Yan Yan, Lixin Wang, Bin Zhou

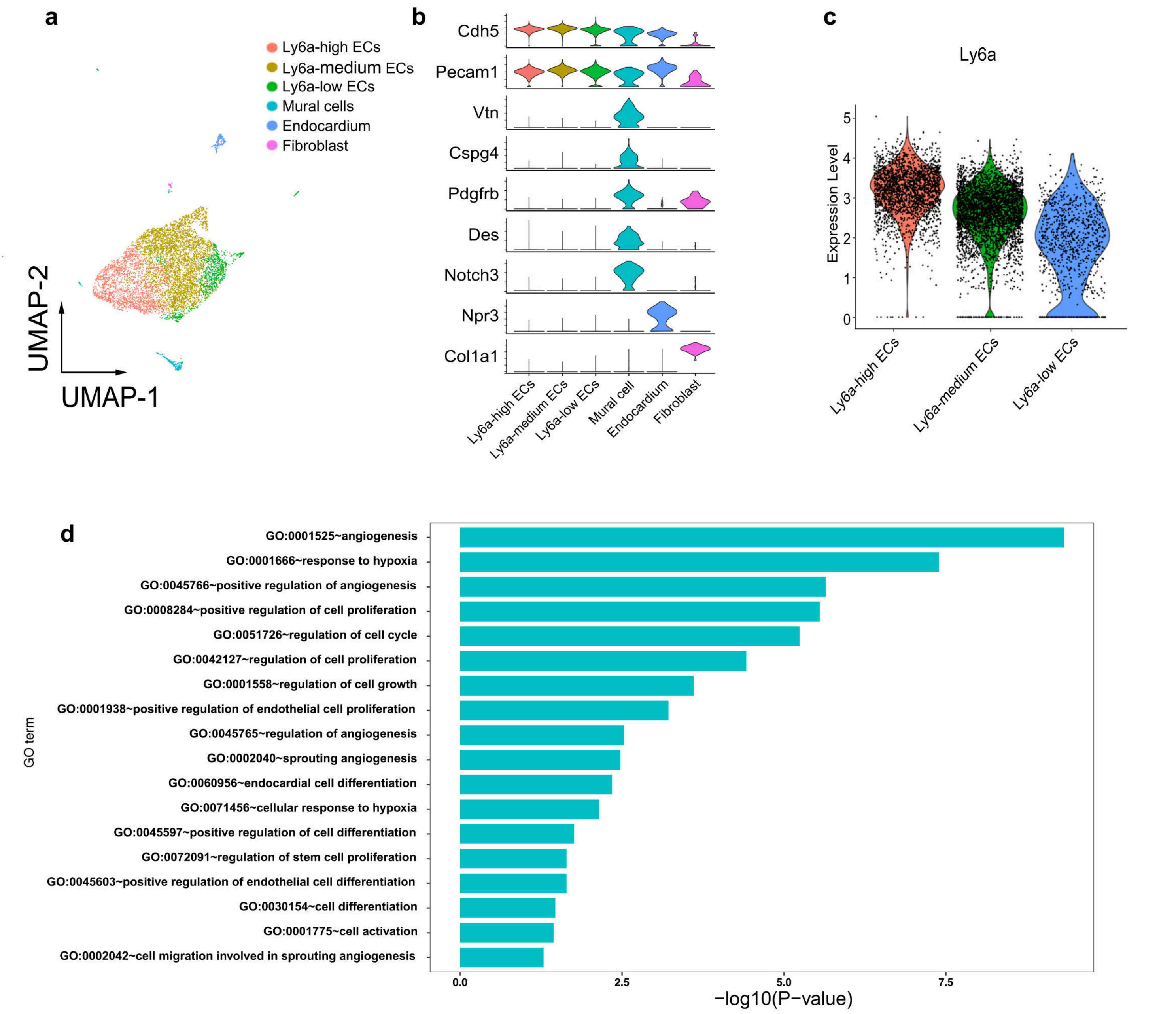

Supplementary Fig. 1 . Single cell RNA-sequencing profiles Sca1^{high} endothelial cells in homeostasis. Single cell RNA-sequencing profiles Ly6a-high endothelial cells in homeostasis. **a**. Visualization of unsupervised clustering of the 7150 cells isolated from heart. 2,000 highly variable genes (HVGs) was selected across all cells using the variance-stabilizing transformation (vst)-based method implemented in the Seurat (v4.0.1) package. We adopted standard principal component analysis (PCA) to the 2,000 HVGs after scaling and centering the normalized data for each gene. To retain enough information, we choose the top 14 principal components to cluster cells and reduce dimensions using FindNeighbors() and RunUMAP() functions with default parameters, while the resolution of 0.6 is selected for FindClusters(). We then annotated clusters with specific cell types according to the expression of marker genes represented in b. **b**.Violin plots shows the expression levels of representative maker genes. **c**. Violin plots shows the expression levels of Ly6a across clusters. The value on the y-axis represent the normalized gene expression. The UMI counts was normalized by library size factor for each cell, which means UMI counts for each cell are divided by the total counts for that cell and multiplied by the scale factor (10,000), then natural-log transformed using log1p. In detail, normalized gene expression values are normalized within each cell as, where is UMI count of gene. **d**. Gene ontology enrichment analysis shows the biological process pathway upregulated in Ly6a-high ECs versus Ly6a-low ECs. The DEGs was calculated by FindMakers() function with 0.3 of logfc. threshold and 0.25 of min.pct. Upregulated 446 DEGs is used for GO:BP enrichment analysis by David (v6.8).

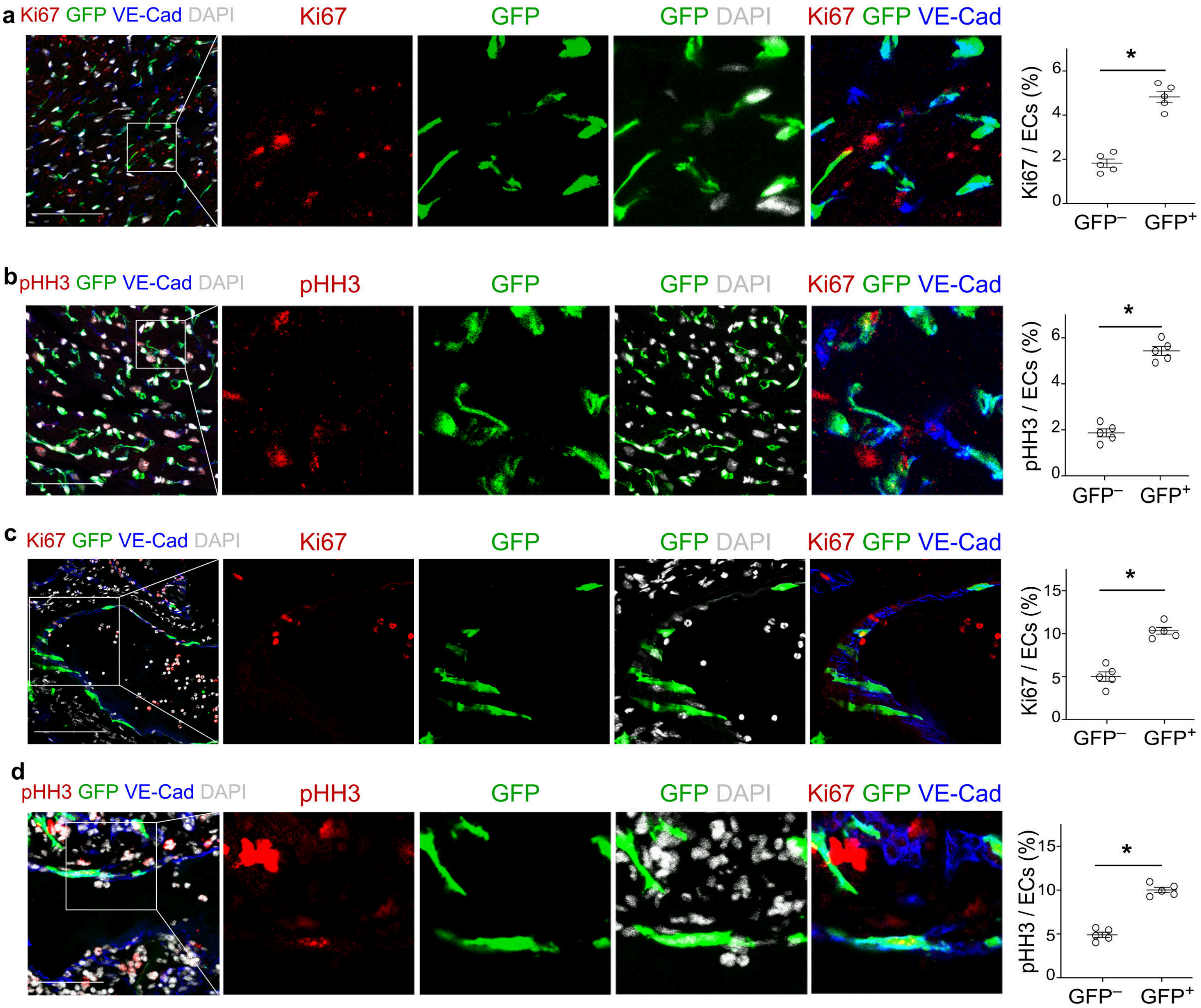

Supplementary Fig. 2. Sca1^{+} endothelial cells increase proliferation after injuries. a, Immunostaining for GFP and VE-Cad, Ki67 on heart sections. Quantification of the percentage of GFP^{-} or GFP^{+} ECs expressing Ki67. **b**, Immunostaining for GFP and VE-Cad, pHH3 on heart sections. Quantification of the percentage of GFP^{-} or GFP^{+} ECs expressing pHH3. **c**, Immunostaining for GFP and VE-Cad, Ki67 on artery sections. Quantification of the percentage of GFP^{-} or GFP^{+} ECs expressing Ki67. **d**, Immunostaining for GFP and VE-Cad, pHH3 on artery sections. Quantification of the percentage of GFP^{-} or GFP^{+} ECs expressing pHH3. Scale bars, 100 μm. Data are mean ± SEM; n = 5; *P < 0.05; n.s., non-significant. Each figure is representative of 5 individual biological samples.
